# Insulin injection knowledge, attitude, and practice of community nurses in a mountainous area of southwest Zhejiang in China: a multi-center cross-sectional study

**DOI:** 10.3389/fendo.2025.1501992

**Published:** 2025-08-18

**Authors:** Xuefen Lan, Simin Zheng, Xiaozhen Ji, Ling Chen, Ling Zhou, Bin Ye, Qingqing Zhu, Xiaojia Zheng, Shunfei Lu

**Affiliations:** ^1^ Department of Medicine, Lishui University, Lishui, China; ^2^ The First Affiliated Hospital of Lishui University, Lishui, China

**Keywords:** diabetes mellitus, insulin injection, community nurses, knowledge, attitude, practice, determinants

## Abstract

**Background:**

Suboptimal insulin injection is widely used to treat Chinese patients with diabetes, with most patients being treated in primary care institutions. However, research on community nurses’ knowledge, attitude, and practice concerning insulin injection in less developed areas of China is extremely scarce.

**Objective:**

To investigate the knowledge, attitude, and practice of community nurses concerning insulin injection in a mountainous area of southwest, Zhejiang, China.

**Methods:**

We employed a cross-sectional study in 30 community healthcare service centers and 1911 randomly selected community nurses between 20th June to 20th July 2023. The Chinese insulin injection knowledge, attitude, and practice questionnaire was used to collect data. Descriptive, correlational, and multivariate linear regression analyses were performed by Stata version 15.0.

**Results:**

In total, 47.7% of nurses had poor insulin injection knowledge, while only 3.7% and 2.5% had poor levels of attitude and practice concerning insulin injection. Sex, location of the institution, working period, marital status, institutional manager, knowledge of the latest guidelines, and undertaking insulin injection training over the last year (all p<0.05) were all identified as independent predictors of insulin injection knowledge. Sex, working period, experience of delivering insulin education to patients, knowledge of the latest guidelines, and undertaking insulin injection training over the previous year (all p<0.05) were identified as independent predictors of insulin injection attitude. Location of the institution, sex, knowledge of the latest guidelines, and undertaking insulin injection training over the last year (all p<0.05) were all independent predictors of insulin injection practice.

**Conclusion:**

Community nurses in this study (Southwest Zhejiang) had relatively good attitudes and practices towards insulin injection, although their specific knowledge was poor. Sex, location of the institution, working period, marital status, knowledge of the guidelines, experience in delivering education, and training experience exhibited significant relationships with the knowledge, attitude, and practice of insulin injection. Therefore, effective tailored, standardized guideline-based training should be recommended to improve the knowledge, attitude, and practice of community nurses regarding insulin injection, especially for married and younger male nurses.

## 1 Introduction

The prevalence, disability and mortality rates of diabetes continue to rise, meaning that this disease has become a major global public health concern (1, 2). China has the largest number of individuals living with diabetes worldwide (approximately 25% of global cases), with a prevalence surging from 7.53% in 2005 to 13.67% in 2023 (3). Diabetes, and its related complications and treatments, have caused severe physiological, psychological, social and economic burdens to patients, families, the medical system, and society. In 2009, China began to implement the National Basic Public Health Service Project and grass-roots medical institutions have assumed the main function of preventing and treating chronic disease; these institutions have become the main driving force for the clinical management of diabetic patients. In 2021, approximately 87% of diabetic patients in China were treated in healthcare institutions at or below the county level (4). Community healthcare providers are now heavily responsible for the prevention and treatment of chronic disease and represent the predominant bodies responsible for the health management of patients with type 2 diabetes. However, the 2018 Report on Chinese Chronic Disease Risk Factor Surveillance showed that the rates of awareness, treatment and control of diabetes in China were only 38.0%, 34.1% and 33.1%, respectively (5). In addition, previous studies have shown that diabetes in China is affecting younger individuals and that the prevalence of diabetes in the rural population is increasing rapidly (2). Therefore, the prevention and control of diabetes in China remains challenging, especially in primary healthcare institutions.

Insulin therapy stands as a critical strategy for achieving glycemic control targets and is widely utilized among diabetic patients (6, 7). For individuals with type 1 diabetes, insulin remains the first-line treatment. In cases of type 2 diabetes, insulin therapy is recommended if glycemic targets are not met following a three-month regimen combining lifestyle interventions and oral hypoglycemic agents (6, 8). For instance, an outpatient survey of type 2 diabetes patients in Sanming City, China, revealed that approximately 44.5% were undergoing insulin therapy (9). Furthermore, a large multicenter cross-sectional survey in China focusing on insulin injection techniques demonstrated that 97.81% of insulin-treated patients had type 2 diabetes, whereas only 2.19% had type 1 diabetes (10). Although China has not implemented a universal free insulin policy, significant cost reductions (exceeding 70% for certain drugs) have been achieved through medical insurance reimbursements and volume-based procurement initiatives (11, 12). Specific regions and vulnerable populations (e.g., low-income residents, elderly island residents) may qualify for free insulin access (13). Nevertheless, substantial economic burdens persist for broader low-income demographics. Beyond financial constraints, other critical issues demand attention with regards to the widespread adoption of insulin therapy. Notable problems include pervasive needle reuse (93.87%) and improper injection site rotation (only 33% performed correctly), contributing to high complication rates such as lipohypertrophy (affecting 48.25% of patients) (10). These practices directly undermine therapeutic efficacy. Consequently, promoting and disseminating standardized insulin injection techniques is imperative (6, 7, 14). Incorrect injection techniques can precipitate complications including subcutaneous lipohypertrophy or lipoatrophy, edema and allergic reactions. These may subsequently lead to suboptimal glycemic control or hypoglycemic events, significantly compromising the therapeutic effectiveness of insulin (15). The 2014–2015 Global Survey of Insulin Injection Techniques indicated limited awareness among healthcare professionals regarding how injection techniques impact glycemic control (6), a finding corroborated by subsequent studies (16–19).

In China, Wu et al. conducted a national cross-sectional study to investigate the knowledge, attitudes, and practices of nurses in China with regard to insulin injection (20). These researchers found that Chinese nurses had a good attitude and practice towards insulin injection, although their knowledge of insulin injection was insufficient. In addition, they demonstrated that knowledge of insulin injection can directly or indirectly influence insulin injection practice through attitude. Because Chinese primary care settings have the majority of chronic patients and are associated with an unmet need for high-quality insulin injection techniques, it is necessary to investigate the knowledge, attitudes, and practices of primary healthcare providers with regard to insulin injection. However, an obvious limitation of this previous study (21) was that the sample cohort predominantly consisted of secondary and tertiary hospitals (99.29%); only 0.71% were primary institutions. The specific situation of primary healthcare providers, especially community nurses, remains unknown. Although other researchers also investigated the knowledge, attitude and practice of nurses towards insulin injection, and the factors that can influence these parameters, in different areas of China, including Liao et al. (22), Zheng et al. (23), Liu et al. (24), these researchers did not focus specifically on community nurses, who remain as the main management force for primary diabetes care in China. Nurses in community healthcare institutions primarily educate DM patients on insulin use, self-management, and lifestyle adjustments, and administer insulin during home visits for vulnerable patients according to prescriptions from the physician (25), especially for patients using insulin for the first time. For these patients, nurses need to thoroughly explain the mechanism of insulin action, the necessity of injection, and common misconceptions (26).

Moreover, while previous studies have considered the levels of knowledge, attitude, and practice of community nurses in relation to insulin injection (20, 22, 27), findings were inconsistent (20, 22). In addition, the sample size of the study reported by Wang et al. (27) was small (a total sample size of 340 nurses with only 63 nurses from community healthcare institutions), and convenient sampling also challenged the reliability of their conclusion and the generalizability of their findings. Finally, most of these previous studies focused on developed areas of China, including Beijing (24), Shanghai (27), and Guangdong (22); few studies have focused on less developed areas or rural areas. Thus, there is still a significant gap in our understanding of the knowledge, attitude, and practice of community nurses in China, especially those from less-developed areas. Therefore, in this study, we investigated the specific knowledge, attitudes, and practices of community nurses with regard to insulin injection, and the factors that can influence these parameters, in less developed areas of China: Lishui City, Zhejiang Province.

## 2 Materials and methods

### 2.1 Study design and setting

This cross-sectional study was conducted at multiple community healthcare service centers across Lishui City in Zhejiang Province of China, between 20th June and 20th July 2023. Lishui City is a less-developed mountainous area of Zhejiang Province and has the lowest Gross Domestic Product (GDP) of the province (21). Approximately 90% of its land is mountains and 47 minorities live in this city. Most inhabitants live in the less developed remote mountainous area, especially the She minority. There are nine counties and 227 healthcare service centers, 181 of these service centers are in rural areas, while 46 are in the urban area of Lishui City (22).

### 2.2 Samples and sampling

This study was conducted in 30 healthcare service centers from three counties and employed cluster random sampling. Economic level (GDP) was used to select the three counties (28). First, a simple random method selected one low, medium, economic level county. Then, ten healthcare service centers were randomly selected from each county, including eight in rural and two in urban areas. All registered community nurses in the selected 30 centers were invited to participate in the investigation. The inclusion criteria were as follows: (a) ≥ 18 years of age; (b) had been hired in a community healthcare institution as a registered nurse; and (c) had performed at least one insulin injection in the previous year. The exclusion criteria were as follows: (a) nurses in training, interns, and nursing students; and (b) nurses who were temporarily in the institution, such as those who were studying or on vacation.

The sample size was calculated as follows: n = z^2^ p (1-p)/d^2^, where α represents the level of significance. When α= 0.05, Z= 1.96; n represents the sample, d represents the allowable error, and P represents the estimated poor knowledge value of the population rate (π). The poor knowledge rate determined by the pre-test survey was approximately 27% (*P* = 0.27; α = 0.05; d = 0.027). We increased the sample size to 15% to account for non-responders, meaning that 1223 healthcare providers were needed. Finally, the survey included 1911 healthcare providers from 30 healthcare service centers in three counties. This study was approved by the ethical review committee of Lishui University (Reference: xx). All participants provided written and informed consent prior to enrollment. The study was conducted in accordance with the Declaration of Helsinki.

### 2.3 Outcome definition and measurement

A 15–20 min self-administrated questionnaire was developed which consisted of two parts: (1) sociodemographic and baseline characteristics, including age, sex, working period, location of institution, educational level, having delivered insulin educations to patients, knowing the latest guidelines of insulin delivery, numbers of insulin injection training in last year. These variables were extracted from related research and guidelines (8, 16, 17, 19, 23), and (2) the Chinese Insulin Injection Knowledge, Attitude, and Practice Questionnaire. Based on the Knowledge, Attitude, and Practices (KAP) model (24) and related guidelines, Wu et al. (16) developed the Chinese Insulin Injection Knowledge, Attitude and Practice questionnaire, consisting of 45 items and three dimensions: knowledge (21 items), attitude (6 items), and practice (18 items). Specifically, the knowledge dimension covers relevant topics such as insulin drugs, injection techniques, and prevention of hypoglycemia; the attitude dimension includes aspects like the importance, standardization, and confidence in insulin injection techniques; and the practice dimension addresses behaviors such as the use of insulin devices and injection techniques. When developing the original questionnaire, Cronbach’s α for insulin injection knowledge, attitude, and practice was 0.686, 0.785, and 0.886, respectively, thus indicating good internal consistency. We also confirmed a satisfactory internal consistency; Cronbach’s α for insulin injection knowledge, attitude, and practice was 0.717, 0.816, and 0.944, respectively. For the insulin injection knowledge dimension, there was 1 point for each item, with a total of 21 points. A total score of <13 points indicated a poor knowledge of insulin injection, 13–17 points were satisfactory, and >17 points were good. In the dimension of insulin injection attitude, 1–5 points were given for items 1–4, and no point was assigned to items 5 and 6. The total score was 20 points; a total score of <12, 12–16, and >16 points indicated poor, satisfactory, and good insulin injection attitude, respectively. In the dimension of insulin injection practice, items 1–14 and 16–18 were given 1 –5 points according to the choice order, and item 15 was given 5–1 points according to the choice order, with a total score of 90 points. A total score of <54 points indicated poor insulin injection practice, 54–72 points was satisfactory and >72 points was good.

### 2.4 Data collection

A total of three data collectors participated in institutional outreach efforts. Data collectors received face-to-face training on ethical liaison protocols with the principal investigator (PI) before the survey. Following ethical approval, the PI and data collectors contacted the directors of participating healthcare centers. During structured meetings, a comprehensive briefing covered: (1) research objectives, (2) questionnaire self-administration procedures, (3) participant eligibility criteria, and (4) data confidentiality protocols. Subsequently, with institutional authorization, QR codes and web links to the online consent form and survey platform (http://www.wjx.cn) were disseminated via designated WeChat groups during June 20–July 20, 2023. All eligible participants in the 30 selected healthcare service centers were invited to participate in the survey autonomously without interviewer involvement. If the participants had any questions about the survey, they were able to contact the researchers by telephone or WeChat. To avoid duplicate entries, we restricted IP access; only one IP address was allowed to complete the survey.

### 2.5 Data analysis

The STATA 15.0 software (Stata Corp. LP, College Station, TX, USA) was used for data analysis. Descriptive statistics, including frequency, percentage, mean, and standard deviation, were used to analyze the sociodemographic and baseline characteristics of the participants. Pearson’s and Spearman’s correlation analyses were used to analyze correlations between knowledge, attitude, and behavior relating to insulin injection. Considering the numbers of sociodemographic and clinical variables, the findings of previous research, and correlation analysis, demonstrated that all independent and dependent variables did not exhibit multicollinearity and were therefore considered as independent variables. The scores for insulin injection knowledge, attitude, and behavior were considered dependent variables. These variables were used to conduct multiple linear regression analysis to investigate the factors that could potentially influence the dimension of insulin injection. *p *< 0.05 was considered statistically significant.

## 3 Results

### 3.1 Sociodemographic and baseline characteristics

In total, 1911 community nurses accepted and completed this online survey (**Table 1**). Approximately 71% of nurses were from rural areas. Of these, 76.8% were female, and most were married (78.2%) and had a bachelor’s degree (73.3%). Half of the nurses were above 35 years of age, with a median age of 37 (30–45) years (range: 18 to 72 years). In total, 48.8% of nurses had worked more than 11 years with a median of 11 (6–20) years. However, only a few of the nurses had a senior title (9.8%) or had a managerial role in their institution (7.1%). When considering all subjects, 68.9% were responsible community nurses, 55.7% had delivered insulin education to patients, and 70.6% knew the latest guidelines associated with the delivery of insulin. In total, 1276 (66.8%) of the respondents had administered insulin over 12 months, 366 (19.2%) had administered insulin over 6 months, and 204 (10.7%) had administered insulin all the time. In terms of training related to the injection of insulin, almost 50% of participants did not receive any form of training over the previous 12 months, 46.5% had received one to three sessions of training, and 3.4% had received more than four sessions of training.

**Table 1:** Socio-demographic and baseline characteristics of the participants.

Variables	Categories	Mean (SD)	Range	n (%)
Locations of the institution	Urban			556(29.1%)
	Rural			1355(70.9%)
Sex	Male			444(23.2%)
	Female			1467(76.8%)
Age (years)	≤25	37.5(9.4)	18-72	151(7.9%)
	26-30			449(23.5%)
	31-35			299(15.6%)
	>35			1012(53.0%)
Responsible community nurses	Yes			1317(68.9%)
	No			594(31.1%)
Work periods (years)	≤3	13.8(9.6)	1-51	254(23.2%)
	4-7			365(19.1%)
	8-11			360(18.8%)
	>11			932(48.8%)
Marital status	Married			1494(78.2%)
	Single			417(21.8%)
Education level	Technical secondary school			116(6.1%)
	Junior college			387(20.3%)
	Bachelor or above			1408(73.7%)
Title of community nurses	Junior			1017(53.2%)
	Intermediate			708(37.0%)
	Associate senior			160(8.4%)
	Senior			26(1.4%)
Managers of the institution	Yes			135(7.1%)
	No			1776(92.9%)
Having delivered insulin education to patients in the last year	Yes			1065(55.7%)
	No			846(44.3%)
Knowing the latest guidelines for insulin delivery	Yes			1349(70.6%)
	No			562(29.4%)
Number of insulin deliveries (person/day)	<8			1707(89.3%)
	≥8			204(10.7%)
Most recent insulin administration	All the time			269(14.1%)
	In a year			1642(85.9%)
Number of insulin injection training in the last year	0			958(50.1%)
	1-3			888(46.5%)
	≥4			63(3.4%)

### 3.2 Insulin injection knowledge score

**Figure 1:**
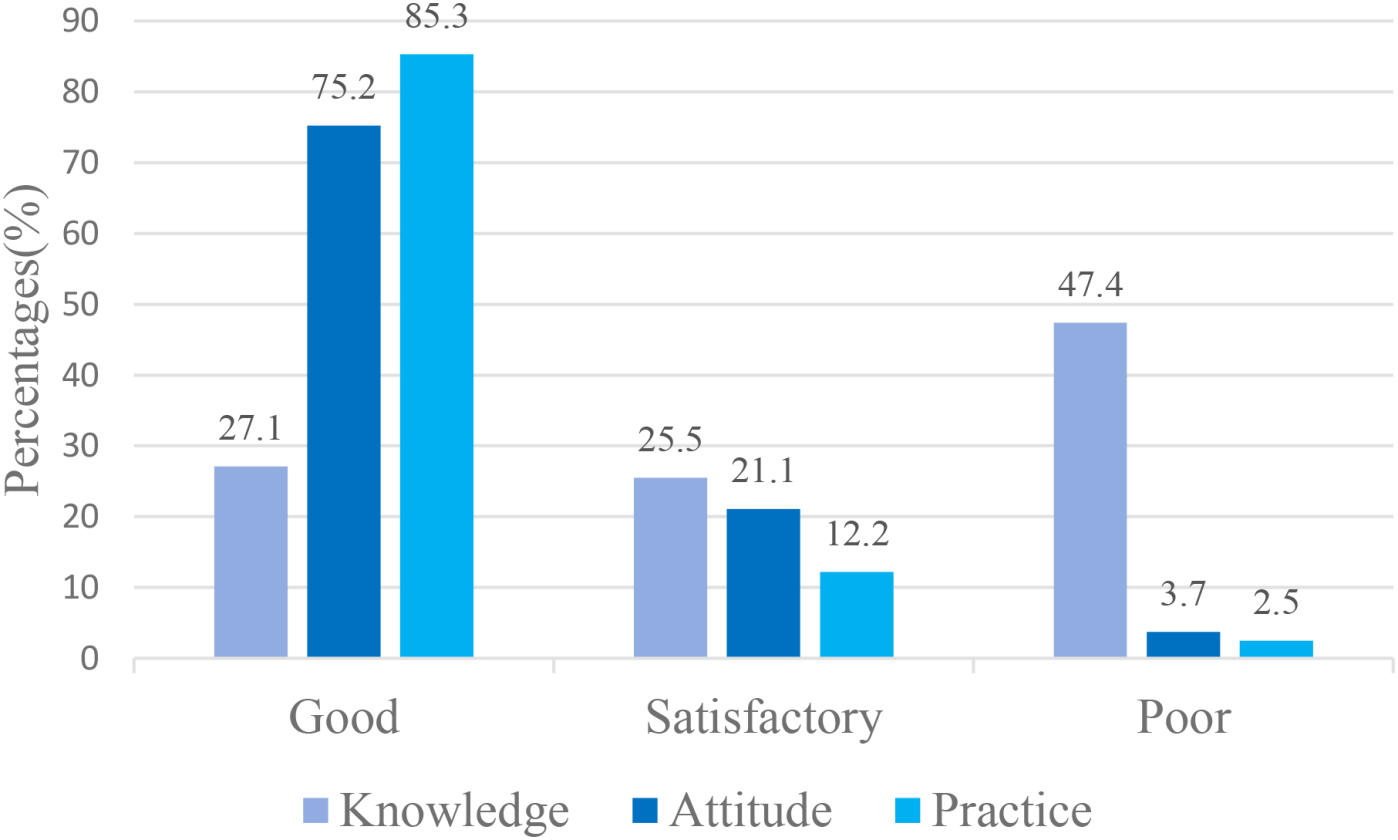
Community nurses’ KAP in insulin injection.

**Figure 2:**
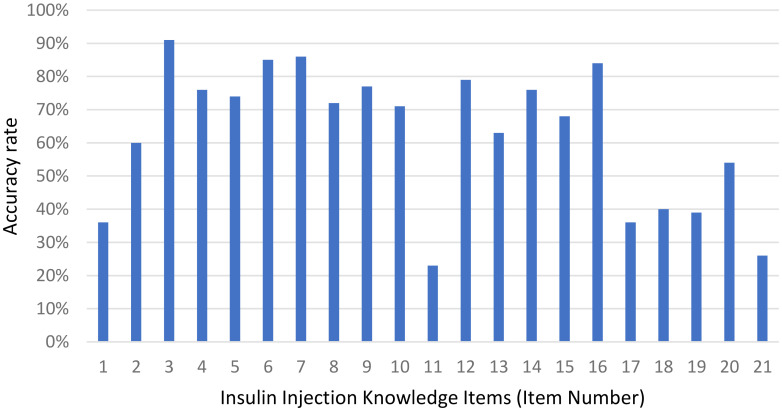
Accuracy rate of the responses to each insulin injection knowledge item among community nurses. Most bars exceed sixty percent, peaking at over eighty percent for items three, six, seven and sixteen, while item eleven falls below twenty-five percent.

The mean insulin injection knowledge score for community nurses was 13.2 ± 4.87; **Figure 1** showed that 27.1% of nurses had a good knowledge score, 25.5% had a satisfactory knowledge score, and 47.4% had a poor knowledge score. Considering the three dimensions of the knowledge score, the mean master basic knowledge score (items 1, 2, 6, 16, 19–21) was 3.85 ± 1.877, the mean master insulin storage knowledge score (items 3–5) was 2.4 ± 0.828, and the mean master insulin injection knowledge score (items 7–15, 17–18) was 6.98 ± 2.765. The mean accuracy rates for the three dimensions were 55%, 80% and 63%, respectively. **Figure 2** shows the overall status of insulin injection knowledge. Items for which less than half of the participants knew included (from low to high): item 11 (the interval between two injections at the same site; 23%), item 21 (hypoglycemia management; 26%), item 1 (types of aspartic insulin; 36%), item 17 (needle disposal method; 36%), item 19 (mixing method for insulin; 39%), and item 18 (injection site administration after withdrawal of needle; 40%).

### 3.3 Insulin injection attitude score

The attitude score for community nurses ranged from 4 to 20, with a mean of 17.0 ± 2.7. **Figure 1** showed that most of the nurses (75.2%) had a good attitude score. Only a few nurses (3.7%) had a poor attitude score, and 21.1% had a satisfactory score. In addition, **Table 2** showed 78.4% of participants considered that the insulin injection technique is important for blood glucose control, 30.7% and 39.9% of participants believed that they could inject insulin in an appropriate manner and were very confident in guiding patients with diabetes to inject insulin correctly, respectively. Moreover, more than 80% of participants were rather or very concerned about the feelings of diabetic patients with regards to insulin injections and the re-use of needles. Approximately 57% of nurses wanted to receive standardized insulin injection training.

**Table 2:** Insulin injection attitude in community nurses (n=1911).

Variables	n(%)
1. Do you think that insulin injection technique is important for plasma glucose control?	
A. Not at all	11(0.6)
B. A little bit	26(1.4)
C. Somewhat	71(3.7)
D. Rather	304(15.9)
E. Very	1499(78.4)
2. Do you think that you can inject insulin properly?	
A. Not at all	114(6.0)
B. A little bit	257(13.4)
C. Somewhat	467(24.4)
D. Rather	486(25.4)
E. Very	587(30.7)
3. How concerned are you about the feeling of diabetic patients at the time of insulin injection?	
A. Not at all	12(0.6)
B. A little bit	62(3.2)
C. Somewhat	263(13.8)
D. Rather	535(28.0)
E. Very	1039(54.4)
4. How concerned are you about needle reuse by diabetic patients?	
A. Not at all	27(1.4)
B. A little bit	71(3.7)
C. Somewhat	243(12.7)
D. Rather	425(22.2)
E. Very	1145(59.9)
5. Are you confident that you can teach diabetic patients to correctly inject insulin?	
A. Not at all	57(3.0)
B. A little bit	125(6.5)
C. Somewhat	395(20.7)
D. Rather	572(29.9)
E. Very	762(39.9)
6. Do you want to receive formal training on insulin injection?	
A. Not at all	29(1.5)
B. A little bit	99(5.2)
C. Somewhat	251(13.1)
D. Rather	435(22.8)
E. Very	1097(57.4)

### 3.4 Insulin injection practice score

The mean insulin injection practice score for the community nurses was 80.7 ± 10.7, with a range of 18 to 90. **Figure 1** showed that more than half of the nurses (85.3%) had a high practice score, and 12.2% had a satisfactory practice score. Very few nurses (2.5%) had a poor insulin injection practice score. The five most commonly performed practices (**Table 3**), including those performed on a regular basis, were washing hands before injection (94.5%), disinfecting the injection site and allowing to dry before injection (94.2%), caring about plasma glucose levels (93.6%), removing air bubbles prior to injection (93%), and setting the appropriate dose of insulin volume before use (93%). However, some practices were performed less commonly, including not injecting insulin when skin induration or swelling was evident (59.4%), leaving an unopened vial of insulin or leaving a full insulin pen at room temperature for 30 min after being removed from a refrigerator (77.1%), leaving the needle subcutaneously for at least 10s following insulin injection (88.4%) and checking tenderness prior to injection (90%).

**Table 3:** Insulin injection practice in community nurses (n=1911).

Variables	n(%)
1. Wash hand before injection	
A. Never	16(0.8)
B. Occasionally	35(1.8)
C. Sometimes	55(2.9)
D. Often	251(13.1)
E. Always	1554(81.4)
2. Unopened vial of insulin or insulin pen fill is left at room temperature for 30 min after being taken out from the refrigerator	
A. Never	152(8.0)
B. Occasionally	86(4.5)
C. Sometimes	200(10.5)
D. Often	328(17.2)
E. Always	1145(59.9)
3. Name, character, expiration date, and remaining volume of insulin in the insulin pen fill is checked before injection	
A. Never	17(0.9)
B. Occasionally	33(1.7)
C. Sometimes	128(6.7)
D. Often	298(15.6)
E. Always	1435(75.1)
4. Full mixing is done before injection of premixed insulin	
A. Never	26(1.4)
B. Occasionally	31(1.6)
C. Sometimes	127(6.6)
D. Often	294(15.4)
E. Always	1433(75.0)
5. Push out air bubbles in the insulin pen or syringe before injecting insulin	
A. Never	22(1.2)
B. Occasionally	23(1.2)
C. Sometimes	89(4.7)
D. Often	259(13.6)
E. Always	1518(79.4)
6. The volume button in the insulin pen is set to the right dose before use	
A. Never	16(0.8)
B. Occasionally	26(1.4)
C. Sometimes	91(4.8)
D. Often	238(12.5)
E. Always	1540(80.5)
7. Ask about meal preparation when giving mealtime insulin	
A. Never	18(0.9)
B. Occasionally	30(1.6)
C. Sometimes	131(6.9)
D. Often	351(18.4)
E. Always	1381(72.3)
8. Pay attention to plasma glucose levels in patients	
A. Never	12(0.6)
B. Occasionally	22(1.2)
C. Sometimes	87(4.6)
D. Often	375(19.6)
E. Always	1415(74.0)
9. Ask about site of last injection	
A. Never	18(0.9)
B. Occasionally	31(1.6)
C. Sometimes	128(6.7)
D. Often	367(19.2)
E. Always	1367(71.5)
10. Ask about injection site tenderness prior to injection	
A. Never	20(1.0)
B. Occasionally	32(1.7)
C. Sometimes	140(7.3)
D. Often	372(19.5)
E. Always	1347(70.5)
11. Injection site is shifted during each injection	
A. Never	15(0.8)
B. Occasionally	30(1.6)
C. Sometimes	100(5.2)
D. Often	395(20.7)
E. Always	1371(71.7)
12. Prior to injection, injection site is carefully examined for skin induration or swelling	
A. Never	17(0.9)
B. Occasionally	29(1.5)
C. Sometimes	106(5.5)
D. Often	339(17.7)
E. Always	1420(74.3)
13. The injection site is disinfected and becomes dry before injection	
A. Never	10(0.5)
B. Occasionally	23(1.2)
C. Sometimes	79(4.1)
D. Often	324(17.0)
E. Always	1475(77.2)
14. Skin pinching technique or entry at an angle of 45° is done when using ≥6 mm insulin pen or syringe	
A. Never	29(1.5)
B. Occasionally	31(1.6)
C. Sometimes	116(6.1)
D. Often	383(20.0)
E. Always	1352(70.7)
15. Insulin is injected despite injection site skin induration or swelling	
A. Never	990(51.8)
B. Occasionally	145(7.6)
C. Sometimes	123(6.4)
D. Often	155(8.1)
E. Always	498(26.1)
16. New needle is used each time insulin is injected	
A. Never	21(1.1)
B. Occasionally	35(1.8)
C. Sometimes	109(5.7)
D. Often	277(14.5)
E. Always	1469(76.9)
17. Needle remains subcutaneously for at least 10 s after insulin is injected	
A. Never	33(1.7)
B. Occasionally	47(2.5)
C. Sometimes	142(7.4)
D. Often	331(17.3)
E. Always	1358(71.1)
18. After insulin is injected, the needle is recapped or is removed using tweezers or needle remover and the needle and syringe are placed in a safe container	
A. Never	50(2.6)
B. Occasionally	31(1.6)
C. Sometimes	114(6.0)
D. Often	296(15.5)
E. Always	1420(74.3)

### 3.5 Correlations between insulin injection knowledge, attitude and practice scores

**Table 4** shows the results of our correlation analysis for insulin injection knowledge, attitude and practice scores in community nurses. Pearson’s correlation analysis showed that there were linear correlations between insulin injection knowledge and attitude (r=0.27, *p*<0.001), between insulin injection attitude and practice (r=0.54, *p*<0.001), and between insulin injection knowledge and practice (r=0.22, *p*<0.001). Spearman’s correlation analysis further confirmed these correlations between insulin injection knowledge, attitude and practice. The strongest correlations were between insulin injection attitude and practice (r=0.40), between insulin injection knowledge and attitude, and between knowledge and practice (r=0.17, *p*<0.001).

**Table 4:** Correlation of the insulin injection knowledge, attitude, and practice scores of community nurses (n=1911).

Pearson’s correlation coefficient					Spearman’s correlation coefficient		
Variables	Correlation value	Knowledge score	Attitude score	Behavior score	Knowledge score	Attitude score	Behavior score
Knowledge score	r	1			1		
	*p*						
Attitude score	r	0.27	1		0.17	1	
	*p*	<0.0001***			<0.0001***		
Behavior score	r	0.22	0.54	1	0.17	0.40	1
	*p*	<0.0001***	<0.0001***		<0.0001***	<0.0001***	

***p<0.001, ∗∗*p <*0.01, ∗*p <*0.05.

### 3.6 Factors affecting the insulin injection knowledge, attitude and practice scores

Multivariable linear regression identified distinct predictors for KAP domains (see **Table 5**). For knowledge, we identified area of institution (β=0.082, 95%CI: 0.371 to 1.381, *p*<0.01), sex (β=-0.049, 95%CI:-2.237 to -1.197, *p*<0.001), working experience (β=0.125, 95%CI:0.230 to 0.872, *p*<0.01), marital status (β=-0.074, 95%CI:-1.462 to -0.285, *p*<0.01), managers of the institution (β=0.052, 95%CI:0.095 to 1.862, *p*<0.05), knowing the latest guideline of insulin delivery (β=0.107, 95%CI:0.615 to 1.670, p<0.001) and the number of insulin injection training sessions in the last year (β=0.078, 95%CI:0.246 to 1.105, *p*<0.01). For attitude, we identified sex (β=-0.059, 95%CI:-0.673 to -0.073, *p*<0.05), working experience (β=0.146, 95%CI:0.170 to 0.544, p<0.001), having delivered insulin educations to patients (β=0.117, 95%CI: 0.353 to 0.917, *p*<0.001), knowing the latest guideline of insulin delivery (β=0.134, 95%CI:0.491 to 1.091, *p*<0.001) and numbers of insulin injection training in last year (β=0.118, 95%CI:0.337 to 0.789, *p*<0.001). For practice, we identified the area of institution (β=-0.047, 95%CI:-2.166 to -0.048, *p*<0.05), sex (β=-0.112, 95%CI:-4.107 to -1.558, *p*<0.001), knowing the latest guideline of insulin delivery (β=0.160, 95%CI: 2.486 to 5.303, *p*<0.001) and the number of insulin injection training sessions in the last year (β=0.096, 95%CI: 0.916 to 2.718, *p*<0.001).

**Table 5:** Factors associated with insulin injection knowledge, attitude, and practice among community nurses (n=1911).

Characteristics	Knowledge		Attitude		Practice	
	β	95% CI	β	95%CI	β	95%CI
Area of institution (urban vs rural)	0.082**	0.371, 1.381			-0.047*	-2.166, -0.048
Sex (male vs female)	-0.149***	-2.237, -1.197	-0.059*	-0.673, -0.073	-0.112***	-4.107, -1.558
Working experience (years)	0.125**	0.230, 0.872	0.146***	0.170, 0.544		
Marital status (married vs single)	-.0.074**	-1.462, -0.285				
Managers of the institution (yes vs no)	0.052*	0.095, 1.862				
Having delivered insulin education to patients (yes vs no)			0.117***	0.353, 0.917		
Knowing the latest guidelines of insulin delivery (yes vs no)	0.107***	0.615, 1.670	0.134***	0.491, 1.091	0.160***	2.486, 5.030
Number of insulin injection training in the last year (≥4 vs 0)	0.078**	0.246, 1.105	0.118***	0.337, 0.789	0.096***	0.916, 2.718
	R^2^ = 0.098, F=15.633		R^2^ = 0.100, F=13.930		R^2^ = 0.081, F=9.067	

***p<0.001, ∗∗*p <*0.01, ∗*p <*0.05.

## 4 Discussion

Our study reveals a critical gap in delivery in that despite positive attitudes (poor attitude only 3.7%), almost half of community nurses (47.7%) had deficient knowledge; this finding is consistent with previous studies (20, 21, 27, 29, 30) but was found to be more pronounced in rural settings. More specifically, the mean level of insulin injection knowledge for community nurses in Lishui was lower than the national level (20), Guangdong province (22), and Anhui province (23), and the proportion of nurses with poor knowledge was also higher than in the areas described previously. Moreover, the mean level of insulin injection practice for community nurses in Lishui was lower than the national level (20), Guangdong province (22), and Beijing City (24), although the mean level of insulin injection practice for community nurses in Lishui was similar to that reported by Zheng et al. (23) and Liao et al. (22). These findings confirmed that the levels of knowledge, attitude, and practice related to insulin injection differ across regions of China. Nurses from primary healthcare institutions had lower levels of knowledge, attitude, and with regard to insulin injection than those in secondary or tertiary institutions (27). These findings highlight that primary healthcare institutions and nursing managers need to pay more attention to community nurses and the improvement of knowledge, attitude and practice related to insulin injections. These community nurses are primarily responsible for the prevention and treatment of diabetes at the grass-roots level; a lack of specialized knowledge, skills, and ability will not only lead to the failure of basic diabetes screening, treatment, nursing and management tasks, but also reduce the trust of residents with regards to the quality of primary care, thus affecting implementation of the National Basic Public Health Service Project; consequently, primary diagnosis and patient triage will be heavily compromised (31). Furthermore, more than half of the community nurses surveyed in Lishui City did not have adequate knowledge of the interval between two injections at the same site, the management of hypoglycemia, types of aspartic insulin, needle disposal methods, mixing methods for insulin, and the treatment of an injection site following needle withdrawal. These findings were similar to those of a previous study (22) and provide direction for future insulin injection training and education for community nurses.

In terms of the attitude of community nurses towards insulin injection training, only 57% of community nurses were willing to receive standardized training for insulin injection; this proportion was lower than the national level (67%) (20). This discrepancy may be due to limited primary care training resources, including teaching, time, and finance, and because the work of community nurses is more focused on chronic disease screening, health education, and vaccination. While the overall practice score was generally adequate, specific deficits were identified in community settings: (1) Injection into indurated/swollen skin sites, (2) Prolonged room-temperature storage (>30 minutes) of opened vials/insulin pens, (3) Premature needle withdrawal (<10 seconds post-injection). These unsafe practices—attributable to knowledge and technique gaps—demand urgent interventions to mitigate risks to patient safety, therapeutic efficacy, and primary care quality. This pattern suggests nurses may execute basic procedures correctly (potentially due to initial training or routine), but lack updated knowledge for full guideline compliance, as evidenced by a Shanghai cross-sectional study: A significant discrepancy persists between clinical practice and current guidelines regarding nurse knowledge, attitude, and practice on insulin injection (32). Thus, implementing practical, hands-on training targeting these suboptimal practices is recommended to address this knowledge-practice gap.

In addition, we identified statistically significant correlations between the knowledge, attitude and behavior scores of community nurses, thus indicating that enhancing insulin injection knowledge and attitude could improve insulin injection practice, highlighting the importance of standard insulin injection interventions or training for community nurses.

To provide tailored and effective interventions or training to improve insulin injection knowledge, attitude and practice, we should also consider the factors that influence these parameters. Male nurses had consistently lower KAP scores, potentially reflecting gendered roles in rural China where females dominate in primary care roles, leading to reduced male exposure to insulin management. These findings concurred with a previous study (33). Training emerged as a pivotal modifiable predictor across all KAP domains, underscoring an urgent need for standardized programs—particularly as 50.1% of participants received no training in the preceding year. Consequently, implementation of evidence-based training guidelines is recommended to enhance insulin injection practices (34). Furthermore, we found that sex can affect insulin injection knowledge, attitude and practice; female community nurses exhibited better performance. A previous study also reported this finding and stated that this may be due to the basic characteristics of females in that they tend to be more careful than males and pay more attention to detail (23). Furthermore, married nurses had better insulin injection knowledge than those who were single; this was consistent with the findings reported previously by Li et al. (35). It may be related to better social support from their partners and families.

Our study revealed significant geographic disparities in insulin injection knowledge and practice among community nurses. Quantitative analysis demonstrated that nurses affiliated with urban healthcare institutions exhibited superior theoretical knowledge, yet paradoxically possessed inferior practical skills compared to their rural counterparts. This dichotomy may be attributed to differential access to continuing education programs. Conversely, the enhanced procedural proficiency observed in rural nurses likely stems from higher patient volume in community care settings, providing greater opportunities for skill reinforcement. This finding aligns with the competency-development paradox described by Zhou et al. in their analysis of skill acquisition patterns among Chinese healthcare professionals (36). With the deepening implementation of the Chinese hierarchical medical system, urban-rural nursing capacity building should shift toward a Context-Adapted Development Paradigm, ultimately achieving systematic evolution from “disparity differentiation” to “functional complementarity”.

The working period was another factor that could influence insulin injection knowledge and attitude. Community nurses who had worked for a longer period had better knowledge and attitude. This finding was consistent with those reported by Zheng et al. (23) and Liao et al. (22). Managers of institutions also had a more positive attitude towards insulin injections. Working for a longer period, or being promoted to managerial level, led to an increase in clinical experience and working ability, while also creating more opportunities to participate in relevant training, thus increasing insulin injection knowledge and attitude.

Another important factor that influences insulin injection attitude is the experience of delivering education to patients. Those who had experience delivering education to patients had a more positive attitude towards insulin injections. This may be related to the fact that health education forces community nurses to systematically integrate knowledge relating to insulin injection, expose their own cognitive contradictions during the process of answering questions from patients, and through self-convincing, reconstruct their technical beliefs (such as deepening the recognition of standardized operation when explaining the impact of injection angle on absorption rate), thereby forming an internal driving force for positive attitudes. In addition, their successful health education experience continuously enhances professional confidence through the ‘ability manifestation-positive feedback’ path of social cognitive theory (37), and then positively transfers to the attitude towards injection practice.

Our findings highlight the development and delivery of standardized training sessions aligned with the latest insulin injection guidelines, focusing on high-risk groups such as unmarried and younger male community nurses. These programs should emphasize practical and hands-on components such as injection site rotation, hypoglycemia management, insulin storage/mixing and needle reuse prevention to address specific knowledge deficits identified in the study (e.g., 47.7% poor knowledge rate). In addition, healthcare institutions and policy makers should establish quarterly or bi-annual refresher workshops incorporating case-based simulations and peer evaluations. This ensures sustained knowledge retention and practice improvement, leveraging the positive attitudes of nurses while mitigating factors, such as limited training experience or rural location disparities, highlighted in the regression analyses.

This study employed a rigorously calculated sample size of 1,911 community nurses across 30 healthcare centers in Southwest Zhejiang, covering diverse economic regions (low, medium and high GDP counties) and both urban/rural settings. The cluster random sampling method ensured proportional representation of the target population (less-developed mountainous areas), addressing a critical gap in existing research that typically focuses on developed regions. This enhances the external validity and generalizability of findings to similar underserved areas in China.

Some limitations of this study should be considered. Firstly, this study was conducted exclusively in Lishui, a mountainous region characterized by economic constraints (the lowest GDP in Zhejiang), ethnic diversity (a minority group settlement area), and geographic isolation (90% mountainous terrain). These factors may limit the direct extrapolation of our findings to other Chinese regions. Therefore, our findings cannot be generalized to other community nurses. Despite regional specificity, our results may reflect challenges faced by nurses in comparable underdeveloped or rural areas of China, such as limited training resources, high patient loads in primary care, and geographic barriers to continuing education. We recommend replicating this study in other underdeveloped provinces (e.g., Guizhou and Yunnan) to assess the transferability of our conclusions. Secondly, the instruments used in this study were self-rated and therefore lacked objectivity. Future studies should aim to combine subjective and objective instruments, including an on-site operation checklist, to acquire stronger evidence relating to insulin injection among community nurses. Thirdly, the relatively low explanatory power of our regression models (R²=0.08-0.10) highlights constraints in capturing the full complexity of insulin injection KAP. This likely stems from unmeasured contextual variables (e.g., institutional training resources, nurse-patient interaction time) and the binary operationalization of some predictors. Crucially, our sampling design (nurses were nested within centers) suggests that multi-level modeling could better disentangle individual- and organizational-level effects. Future investigations should adopt Structural Equation Modeling (SEM) or multi-level analysis to incorporate center-specific variables (e.g., rural/urban resource disparities) and explore cross-level interactions.

## 5 Conclusion

Community nurses in Southwest Zhejiang demonstrated suboptimal knowledge regarding insulin injection techniques and management despite generally positive attitudes and practices. To address this identified deficiency, we propose the implementation of mandatory, evidence-based training programs aligned with current clinical guidelines, with prioritization given to identified high-risk subgroups (e.g., male, newly employed nurses). Furthermore, healthcare institutional administrators and policy makers should leverage telehealth platforms and mobile supervision systems to mitigate urban-rural disparities in knowledge accessibility during this digital transformation era.

Original Research article.

## Data Availability

The raw data supporting the conclusions of this article will be made available by the authors, without undue reservation.
