# Supplementary material for: Insulin injection knowledge, attitude, and practice of community nurses in a mountainous area of southwest Zhejiang in China: a multi-center cross-sectional study
Source: Front Endocrinol (Lausanne). 2025 Aug 18;16:1501992. doi: 10.3389/fendo.2025.1501992 (PMC12399374; doi:10.3389/fendo.2025.1501992)
Supplement: Supplementary file 1 [file Table1.docx]

**Appendix A**

**中文版护士胰岛素注射知信行问卷**

| **第一部分 胰岛素注射知识问卷**  此部分旨在了解护理人员胰岛素注射方面的知识情况，请您在您认为合适的项目内打“🗸”。 |
| --- |
| 1. 门冬胰岛素属于下列哪一种类？ 2. 长效胰岛素 3. 短效胰岛素 4. 速效胰岛素 5. 中效胰岛素 |
| 2. 短效胰岛素注射的注射时间？  A. 餐后  B, 餐前30分钟  C. 餐前5-10分钟  D. 餐前即刻 |
| 3. 未开封的胰岛素正确的储藏温度是多少？  A. 10–15 ℃  B. 15–30 ℃  C. 2–8 ℃  D. <0 ℃ |
| 4. 胰岛素从冰箱冷藏中取出，需要复温多长时间?  A. 15 min  B. 30 min  C. 45 min  D. 60 min |
| 5. 已开启胰岛素在室温下可以保存多长时间?   1. 15 天 2. 30 天 3. 45 天 4. 60 天 |
| 6. 目前使用的胰岛素注射笔和注射器，最安全的注射针头长度为？  A. 4 mm and 6 mm  B. 4 mm and 8 mm  C. 5 mm and 6 mm  D. 5 mm and 8 mm |
| 7. 胰岛素注射应该用何消毒剂消毒皮肤?  A. 安尔碘  B. 70-80%乙醇  C. 碘伏  D. 含氯消毒剂 |
| 8.人体合适的注射胰岛素的部位有哪些？  A. 腹部  B. 大腿外侧  C. 臀部外上侧  D. 上臂外侧 |
| 9. 常规胰岛素在不同注射部位的吸收速度由快到慢的是？  A. 大腿，腹部，上臂，臀部  B. 腹部，上臂，大腿，臀部  C. 腹部、臀部、上臂、大腿  D. 上臂，腹部，大腿，臀部 |
| 10. 在胰岛素注射部位轮换过程中，每次注射点应与上次注射点至少间隔多少距离？  A. 1 cm  B. 2 cm  C. 3 cm  D. 4 cm |
| 11. 在胰岛素注射时，应尽量避免在多长时间内重复使用同一注射点？  A. 1周  B. 2周  C. 3周  D. 4周 |
| 12. 注射胰岛素时正确的捏皮方法是？  A. 拇指、无名指捏  B. 拇指、中指捏  C. 拇指、中指与食指  D. 五个手指一起捏起 |
| 13. 使用较短（4mm或5mm）的针头注射时，采用的进针角度是？  A. 30°  B. 45°  C. 60°  D. 90° |
| 14. 使用较长（≥6mm）的针头注射时，除可捏皮外，还可以采用的进针角度是？  A. 15°  B. 30°  C. 45°  D. 60° |
| 15. 使用胰岛素笔注射，推注完毕后，针头应停留多长时间?  A. 2–3 秒  B. 6–9 秒  C. ≥ 10 秒  D. 不停留 |
| 16. 与胰岛素注射相关的并发症有哪些？  A. 皮下脂肪增生或萎缩  B. 感染  C. 疼痛  D. 出血和淤血 |
| 17. 使用胰岛素笔注射完毕后，处理针头的正确方法是？  A. 单手回套外针帽，置锐器盒  B. 回套內针帽，置锐器盒  C. 用专用取针器或镊子取下针头，置锐器盒  D. 回套针帽，留在胰岛素笔上 |
| 18. 应用胰岛素笔注射完毕拔出针头后，注射部位应？  A. 干棉签按压  B. 酒精棉签按压  C. 手指按压  D. 无需按压 |
| 19. 以下关于胰岛素混匀方法错误的是：  A. 肉眼检查确认药液为白色均匀的混悬液  B. 如果药液仍然未充分混匀，则重复操作  C. 在室温下5s内双手水平滚动胰岛素笔芯10次，然后10s内上下翻转10次  D．中效胰岛素与短效胰岛素混合时，应先抽中效胰岛素，再抽短效胰岛素 |
| 20.以下关于胰岛素专用注射器的说法错误的是：  A. U-100胰岛素注射器只适用于抽取浓度为100 U/ml的胰岛素  B. U-40胰岛素注射器不应抽吸胰岛素笔芯内药液进行注射  C. U-40胰岛素注射器可用于抽吸胰岛素笔芯内药液  D. U-40胰岛素注射器只适用于抽吸浓度为 40 U/ml的胰岛素 |
| 21. 以下关于低血糖的处理，错误的是：  A. 怀疑低血糖时立即检测血糖，以明确诊断，无法测血糖时暂时按低血糖处理  B. 意识清楚者给予5%葡萄糖液或10%葡萄糖液静脉注射  C. 意识清楚者口服15 - 20g糖类食品（葡萄糖为佳）  D. 意识障碍者给予50%葡萄糖液20ml静脉注射 |
| **第二部分 胰岛素注射信念问卷**  此部分旨在了解护理人员对胰岛素注射相关知识的信念情况，请您在您认为合适的项目内打“🗸”。 |
| 1. 您认为胰岛素注射技术对血糖控制的影响很重要吗？  A. 一点也不  B. 有一点  C. 有些  D. 相当  E. 非常 |
| 2. 您认为自己能够正确执行胰岛素注射操作技术规范吗?  A. 一点也不  B. 有一点  C. 有些  D. 相当  E. 非常 |
| 3. 您关注糖尿病患者对注射胰岛素的感受吗?  A. 一点也不  B. 有一点  C. 有些  D. 相当  E. 非常 |
| 4. 您关注糖尿病患者注射针头的重复使用吗?  A. 一点也不  B. 有一点  C. 有些  D. 相当  E. 非常 |
| 5. 指导糖尿病患者进行胰岛素注射，您对自己的教育能力有信心吗?  A. 一点也不  B. 有一点  C. 有些  D. 相当  E. 非常 |
| 6. 您需要参加胰岛素注射的相关培训?  A. 一点也不  B. 有一点  C. 有些  D. 相当  E. 非常 |
| **第三部分 胰岛素注射行为问卷**  此部分旨在了解护理人员在临床工作过程中对糖尿病患者胰岛素注射处理的实践情况，请您根据实际情况填写，在相应的选项上打“🗸”。 |
| 1. 给患者注射胰岛素前洗手   A. 从来没有  B. 偶尔  C. 有时  D. 经常  E. 总是 |
| 2. 对未开封的瓶装胰岛素或胰岛素笔芯提前30min从冰箱中取出回暖  A. 从来没有  B. 偶尔  C. 有时  D. 经常  E. 总是 |
| 3.胰岛素注射前，检查笔芯药液的名称、性状、有效期、余量  A. 从来没有  B. 偶尔  C. 有时  D. 经常  E. 总是 |
| 4. 在注射预混胰岛素前进行充分混匀  A. 从来没有  B. 偶尔  C. 有时  D. 经常  E. 总是 |
| 5. 在使用注射胰岛素笔或注射器执行胰岛素注射前排气   1. 从来没有 2. 偶尔 3. 有时 4. 经常 5. 总是 |
| 6. 在使用胰岛素笔注射前将胰岛素笔剂量旋钮旋至所需刻度   1. 从来没有 2. 偶尔 3. 有时 4. 经常 5. 总是 |
| 7. 给患者注射胰岛素时，询问患者备餐情况   1. 从来没有 2. 偶尔 3. 有时 4. 经常 5. 总是 |
| 8. 给患者注射胰岛素前，关注患者血糖情况   1. 从来没有 2. 偶尔 3. 有时 4. 经常 5. 总是 |
| 9. 给患者注射胰岛素前，询问患者上一次注射部位   1. 从来没有 2. 偶尔 3. 有时 4. 经常 5. 总是 |
| 10. 给患者注射胰岛素前，询问患者注射部位有无疼痛感   1. 从来没有 2. 偶尔 3. 有时 4. 经常 5. 总是 |
| 11. 根据轮换法选择注射部位   1. 从来没有 2. 偶尔 3. 有时 4. 经常 5. 总是 |
| 12. 给患者注射胰岛素前，仔细评估注射部位是否有皮下硬结、肿胀等   1. 从来没有 2. 偶尔 3. 有时 4. 经常 5. 总是 |
| 13. 给患者注射胰岛素前，均会消毒注射部位皮肤且自然待干后注射   1. 从来没有 2. 偶尔 3. 有时 4. 经常 5. 总是 |
| 14. 使用≥6mm的笔用针头或注射器注射胰岛素时，使用捏皮技术或采用倾斜45°进针   1. 从来没有 2. 偶尔 3. 有时 4. 经常 5. 总是 |
| 15. 在患者皮肤硬结、肿块上注射过胰岛素   1. 从来没有 2. 偶尔 3. 有时 4. 经常 5. 总是 |
| 16. 给患者注射胰岛素时，每次使用新的针头   1. 从来没有 2. 偶尔 3. 有时 4. 经常 5. 总是 |
| 17. 在使用胰岛素笔注射时，胰岛素推注完后把挣钱，针头停留至少10秒   1. 从来没有 2. 偶尔 3. 有时 4. 经常 5. 总是 |
| 18. 给患者注射胰岛素完毕单手回套外针帽后旋下针头，或用专用取针器或镊子去下针头，置锐器盒   1. 从来没有 2. 偶尔 3. 有时 4. 经常 5. 总是 |
